# Mapping body-building potential

**DOI:** 10.7554/eLife.14830

**Published:** 2016-03-07

**Authors:** Aida Rodrigo Albors, Kate G Storey

**Affiliations:** Division of Cell & Developmental Biology, School of Life Sciences, University of Dundee, Dundee, United Kingdom; Division of Cell & Developmental Biology, School of Life Sciences, University of Dundee, Dundee, United Kingdomk.g.storey@dundee.ac.uk

**Keywords:** neuromesodermal progenitors, wnt/β-catenin signalling, primitive streak, lateral mesoderm, axis elongation, Mouse

## Abstract

Experiments in mice shed new light on an elusive population of embryonic cells called neuromesodermal progenitors.

**Related research article** Wymeersch FJ, Huang Y, Blin G, Cambray N, Wilkie R, Wong FCK, Wilson V. 2016. Position-dependent plasticity of distinct progenitor types in the primitive streak. *eLife* **5**:e10042. doi: 10.7554/eLife.10042

**Image** Putative neuromesodermal progenitors (green) in a mouse embryo

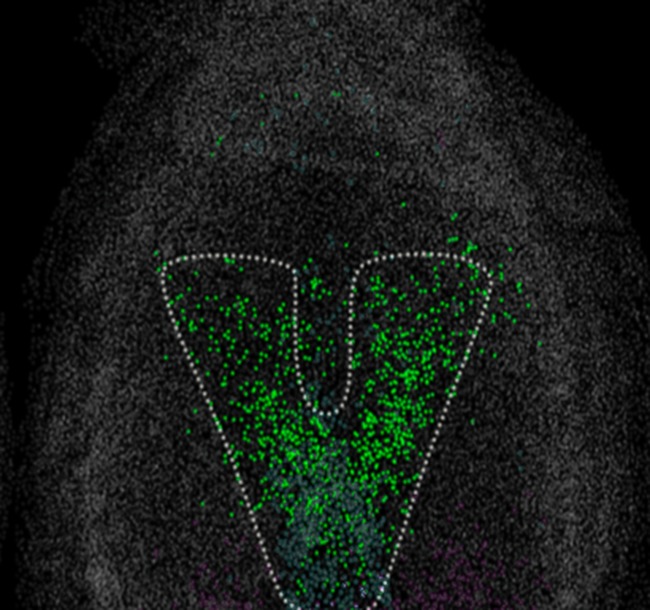

Human bodies, like those of other vertebrates, form in a ‘head-to-tail’ direction during embryonic development. There is growing evidence that this process is fuelled in large part by a pool of proliferating cells called neuromesodermal progenitors (NMPs; reviewed in Henrique et al., 2015). These cells have been found in zebrafish, chick and mouse embryos, and also in human embryos (Olivera-Martinez et al., 2012), and they seem to produce both the neural tissue that makes the spinal cord and mesodermal tissues such as muscle and bone.

The breakthrough evidence for the existence of NMPs came from researchers painstakingly tracing the descendants of individual cells in mouse embryos (Tzouanacou et al., 2009). Other studies suggest that NMPs occur in and around the the primitive streak, a structure which is found at the tail-end of early embryos (Cambray and Wilson, 2007).

Until recently we knew little about the dynamics of the NMP cell population. It was even unclear which precise regions of the embryo had the potential to be NMPs. Did specific cells become NMPs early in development? Or was the NMP potential conferred on the cells by external signals that continuously define an NMP microenvironment or niche? Now, in eLife, Valerie Wilson and co-workers from the MRC Centre for Regenerative Medicine – including Filip Wymeersch as first author – report new insights into the elusive NMPs (Wymeersch et al., 2016).

Wilson, Wymeersch and co-workers set out first to define the potential of cells in and around the primitive streak in early mouse embryos. They found that the cells at, or near to, the end of the primitive streak that is closest to the embryo’s head were restricted to neural and mesodermal fates. The cells nearer the tail-end of the primitive streak were instead restricted to adopt mesodermal fates.

Wymeersch et al. then went on to track putative NMPs as the embryos developed. There isn’t yet a specific marker for NMPs, so they looked for cells that co-expressed markers of early neural and mesoderm cells (Figure 1). The number of cells that expressed both of these markers peaked in embryos that were nine and a half days old and then decreased, which is consistent with previous reports based on analyses of the fates of single cells (Tzouanacou et al., 2009).

**Figure 1.**
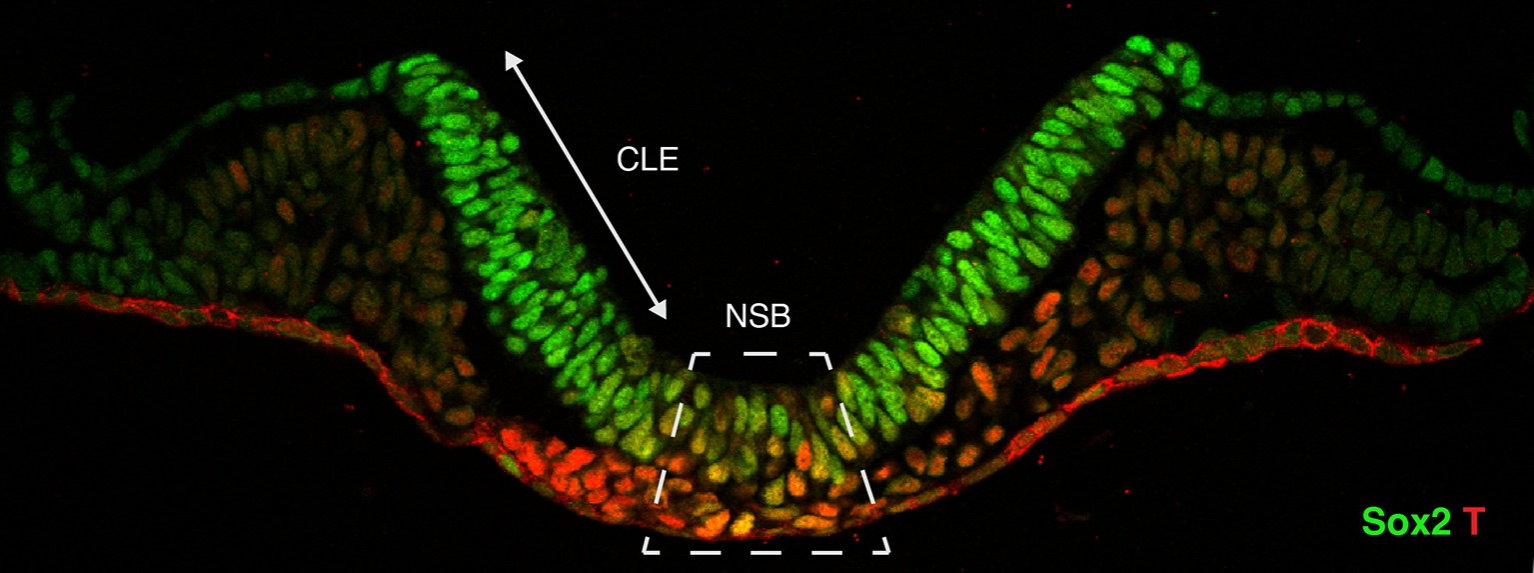
Cross-section through the primitive streak of an early mouse embryo. Neuromesodermal progenitors are embryonic cells that are thought to produce both neural and mesodermal tissues. Sox2 (shown in green) is a marker of early neural cells, while T (red) is a marker of early mesoderm cells. Putative neuromesodermal progenitors co-express these markers and appear yellow. Wymeersch et al. found cells co-expressing Sox2 and T in and around the primitive streak, in the so-called node-streak border (NSB) and caudal lateral epiblast (CLE). Image credit: Rodrigo Albors and Storey.

Further examination revealed that the levels of these two markers varied considerably among cells of the tail-most end of the embryo. Wymeersch et al. then found that cells with higher levels of the early neural marker were biased towards neural fates, and vice versa for the early mesoderm marker.

Wymeersch et al. went on to ask if these differences were fixed, or whether they could be changed by external signals. To answer this question, they transplanted small groups of cells from one region of an embryo into another region. These highly skilled experiments revealed that these cell fate biases were not hardwired but instead depended on external signals or cues and could be changed.

The Wnt/β-catenin signalling pathway has recently been implicated in regulating the fate of NMPs, and particularly in promoting mesoderm fate (Martin and Kimelman, 2012; Garriock et al., 2015; Jurberg et al., 2014). However, in these experiments, the signalling pathway was manipulated in more than just the NMP cell population. Wymeersch et al. used their ability to transplant precise populations of cells to ask if Wnt/β-catenin signalling is required in NMPs. They confirmed that NMPs absolutely needed β-catenin activity to adopt a mesoderm fate, and discovered a new role for Wnt/β-catenin signalling in expanding and maintaining the NMP population in growing mouse embryos.

Last, Wymeersch et al. turned their attention to the population of cells that strongly expressed early mesodermal markers at the tail-most end of the embryo. This region is fated to become mesoderm tissue that makes up the sides and underside of the animal’s body. By transplanting these cells into the NMP niche, Wymeersch et al. found that this population of cells could not be re-directed to become NMPs; instead, these cells remained committed to a mesodermal fate. Furthermore, this was even the case when the cells were made to express a gene that controls early neural development. Wymeersch et al. named this newly identified cell population “lateral/paraxial mesoderm progenitors”, or LPMPs for short, and also showed that their differentiation into mesoderm cell types did not depend on β-catenin.

The NMP field is advancing rapidly, and many recent studies have revealed new details (Tsakiridis and Wilson, 2015; Turner et al., 2014; Gouti et al., 2014; Denham et al., 2015). However, to be confident that NMPs exist as individual entities in living embryos, there is still one key experiment missing. This is the demonstration that a single cell in an embryo that co-expresses markers of early neural and mesoderm cells can indeed contribute to both neural and mesodermal tissues.

Other important questions remain. For instance, what signals does the NMP niche provide to maintain the ability of cells to produce both neural and mesodermal tissues? What signals, in addition to Wnt, also come to influence the fate of an NMP? Finding these signals may have implications for biomedical research, or even personalized medicine, since it will allow us to direct NMPs to make neural or mesodermal tissue in the laboratory.

Moreover, with the advance of single-cell technologies, we must soon be able to revisit NMPs at a single-cell resolution. This will aid in uncovering more about the NMP cell population and provide a better understanding of the newly identified LPMP cell type. Identifying genes that are uniquely expressed in NMPs will also be key to generating new tools to manipulate gene expression specifically in this cell population.
